# Improvement studies for equitable and evidence-based innovation: an overview of the ‘IM-SEEN’ model

**DOI:** 10.1186/s12939-023-01915-5

**Published:** 2023-06-17

**Authors:** Luke N. Allen, Oathokwa Nkomazana, Sailesh Kumar Mishra, Michael Gichangi, David Macleod, Jacqueline Ramke, Nigel Bolster, Ana Patricia Marques, Hilary Rono, Matthew Burton, Min Kim, Bakgaki Ratshaa, Sarah Karanja, Ari Ho-Foster, Andrew Bastawrous

**Affiliations:** 1 London School of Hygiene & Tropical Medicine (LSHTM), Keppel St, London, WC1E 7HT UK; 2 University of Botswana, Gaborone, Botswana; 3 Nepal Netra Jyoti Sangh, Kathmandu, Nepal; 4 Kenyan Ministry of Health, Nairobi, Kenya; 5 Peek Vision and LSHTM, Berkhamsted, UK; 6 Kitale Hospital and Peek Vision, Kitale, Kenya; 7 KEMRI, Nairobi, Kenya

**Keywords:** Equity, Continuous improvement, Universal Health Coverage

## Abstract

**Background:**

Health inequalities are ubiquitous, and as countries seek to expand service coverage, they are at risk of exacerbating existing inequalities unless they adopt equity-focused approaches to service delivery.

**Main text:**

Our team has developed an equity-focused continuous improvement model that reconciles prioritisation of disadvantaged groups with the expansion of service coverage. Our new approach is based on the foundations of routinely collecting sociodemographic data; identifying left-behind groups; engaging with these service users to elicit barriers and potential solutions; and then rigorously testing these solutions with pragmatic, embedded trials. This paper presents the rationale for the model, a holistic overview of how the different elements fit together, and potential applications. Future work will present findings as the model is operationalised in eye-health programmes in Botswana, India, Kenya, and Nepal.

**Conclusion:**

There is a real paucity of approaches for operationalising equity. By bringing a series of steps together that force programme managers to focus on groups that are being left behind, we present a model that can be used in any service delivery setting to build equity into routine practice.

## Background: pervasive health inequalities

Health outcomes are inequitably distributed across and between populations [1–3]. The inverse care law states that the availability of medical care is inversely proportional to need [4]. The most disadvantaged groups in society often experience the worst health outcomes [5].

As signatories to the Sustainable Development Goals seek to advance Universal Health Coverage (UHC), governments and health system leaders face complex decisions about how to extend access to services whilst balancing equity considerations against cost-effectiveness: for example, it is often expensive to reach disadvantaged and remote communities.

In the 2010 review ‘Fair society, Healthy Lives’, Michael Marmot introduced the concept of ‘proportionate universalism’ (Table 1), arguing that health services should benefit all, but with the greatest gains experienced by those with the greatest needs [1]. Following on from this, in 2014, WHO published ‘Making fair choices on the path to UHC’ which urged system leaders to focus on extending coverage of a core basket of priority services to all citizens; paying particular attention to ensuring that disadvantaged groups are not left behind [6]. In the same year, WHO and the World Bank issued a joint call for services to routinely gather data on core sociodemographic indicators, arguing that data collection is the essential first step in moving towards redressing health inequalities [7].

**Table 1:** Proportionate universalism [1, 8, 9]

Proportionate universalism combines targeting with universalist principles of equality and fairness; seeking to provide services to all, with additional resources provided to members of specific groups who face structural disadvantage [1]. This builds on prioritarian [8] principles outlined in the Alma-Ata Declaration that calls for “the progressive improvement of comprehensive health care for all… Giving priority to those most in need”[9].	

Unfortunately, whilst sociodemographic data collection has become more widespread, ubiquitous inequalities persist, [3] suggesting that our health systems are not translating new intelligence into meaningful action. An added problem is that interventions and service modifications designed to address inequalities are rarely evaluated using robust scientific techniques such as randomised controlled trials (RCTs) [10].

Our team – a collaboration between the International Centre for Eye Health (ICEH) at the London School of Hygiene & Tropical Medicine (LSHTM), the University of Botswana, the Kenyan Ministry of Health, Nepal Netra Jyoti Sangh, the College of Ophthalmology for Eastern, Central and Southern Africa, and Peek Vision – has been funded by the NIHR and The Wellcome Trust to develop and field-test an equity-focused continuous improvement model that addresses these challenges (Table 2). Whilst other publications from our group provide detailed methods for each of the elements and will present emerging findings, this paper seeks to provide a holistic overview of how the model fits together, the issues it seeks to address, and potential application to other fields.

**Table 2:** Applying the model in the field of eye care

Whilst the model has been designed so that it can be applied in any setting, our focus is improving equitable use of primary care services in line with the broader aims of Universal Health Coverage. Our group is in the process of field-testing the model in large community-based eye screening programmes operating in Botswana, India, Kenya and Nepal	
Eye health is a major global public health issue and 90% of the 1.1 billion people with correctable vision impairment live in low and middle income countries [11]. It is thought that only around half of those identified with a need at screening actually attend clinic to receive treatment – which is close to the African regional mean for non-attendance across all service types [12]. Evidence is limited, but suggests that women, widows, and those from rural areas are the least likely to receive the care they need [11, 13]	
The advent of smartphone-based eye assessment and the digitisation of vision screening programmes has made it much more affordable to rapidly screen and treat large populations. The most widely used digital platform is currently supplied by Peek; a social enterprise non-profit spin-out from LSHTM whose app-based programme has been rigorously evaluated [14–20]. Peek has agreements in place with international non-governmental organisations (NGOs), local NGOs and governments in twelve LMICs to support eye screening programmes that should reach tens of millions of people over the next decade [21]. Our group has been working with Peek to embed the IM-SEEN model into their processes and software. We anticipate that this method will allow local eye health system leaders to conduct rapid randomised controlled trials (RCTs) within their programmes to test incremental modifications aimed at reducing socioeconomic gaps in service provision, with the greatest gains seen in disadvantaged groups	

### The IM-SEEN model

**Fig. 1:**
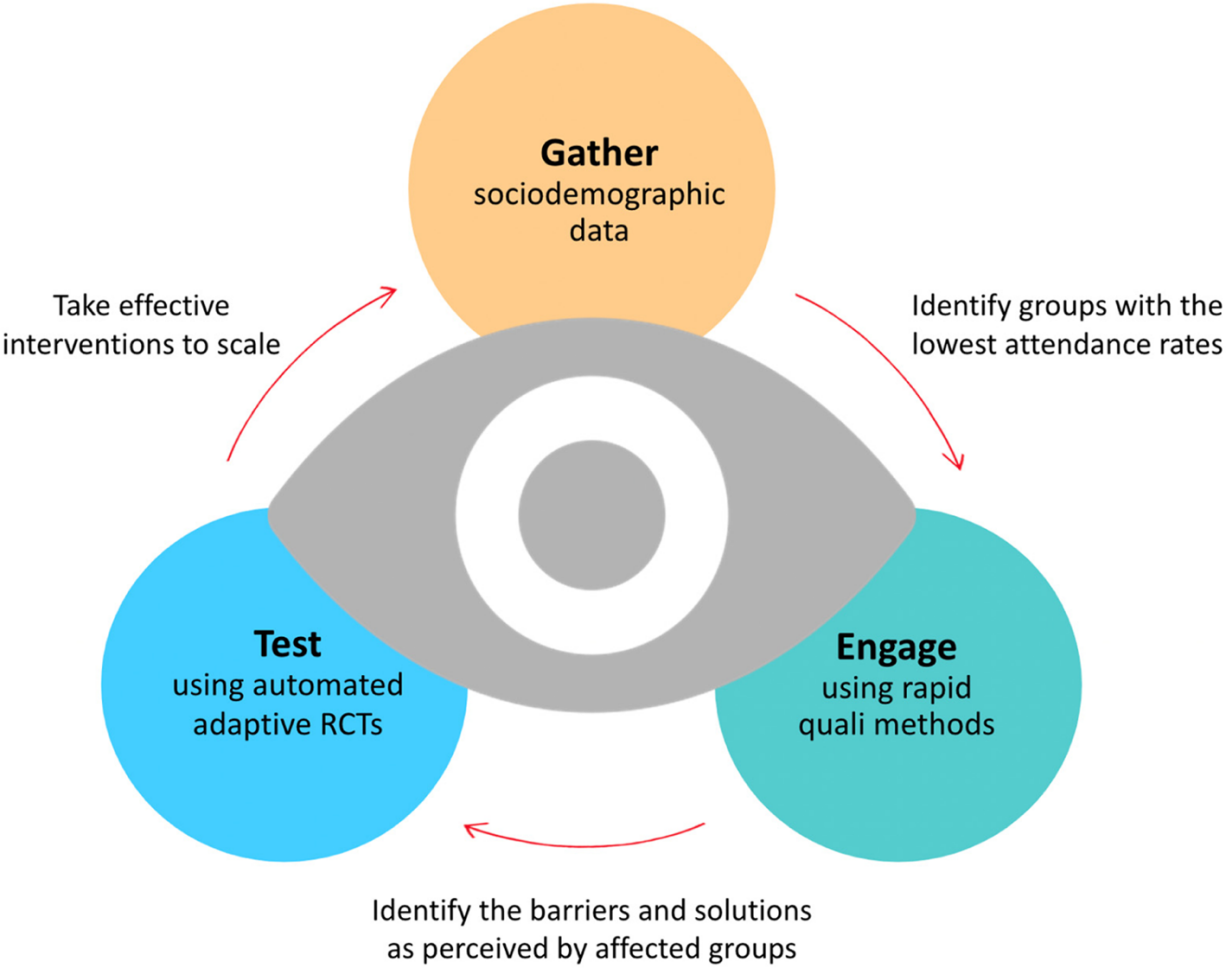
The IM-SEEN approach to continually improving equitable outcomes

The model that we have developed is based around three elements: routinely gathering sociodemographic data from service users and regularly interrogating these data to identify which groups are experiencing the worst outcomes; engaging with representatives from these groups to elicit their perspective on the main issues and solutions; and then using rigorous randomisation-based testing of these potential solutions in order to equitably improve outcomes (Fig. 1). Each element requires scientifically-grounded work; gathering and analysing data; conducting interviews; and running pragmatic embedded trials.

We have dubbed the overall approach ‘IM-SEEN’: Improvement Studies for Equitable and Evidence-based Innovation. The acronym highlights our focus on engaging with members of underserved groups and basing the improvement cycle around their concerns and ideas, rather than making assumptions or acting on the behalf of these communities.

The IM-SEEN model was iteratively developed by a team of public health specialists, statisticians, qualitative researchers, economists, programme implementers, ethicists and government policymakers. AB, ON, MG, SM, MB and NB scoped the initial need for an approach to continually improving health service outcomes with a focus on those ‘left behind’ to close socioeconomic gaps. LA led a series of reviews and the drafting of early models which were iteratively refined between 2021–2023 during a series of online and in-person workshops funded by the NIHR and Wellcome Trust. The core team are co-authors of this paper.

### The IM-SEEN process for continuous equitable improvement

#### Gathering sociodemographic data to identifying underserved groups

The first step in model involves quantifying baseline inequalities and identifying the sociodemographic group(s) with the worst outcomes. This process should be built into routine data collection, with analysis and reporting automated as much as possible.

In our eye programmes, screeners are digitally documenting the sociodemographic characteristics (including age, sex, ethnicity/language, religion, education, health status, assets, and income) of every individual who is found to have an eye need and referred on to receive further care. Quarterly meetings are used to review these data with the programme leads. We use multivariable logistic regression to identify which characteristics are most strongly associated with non-attendance. Detailed methods are available in a separate publication [22].

#### Understanding why certain groups do not attend – and what could be done about it

Once the characteristics most strongly associated with non-attendance have been identified, the next step is to engage with representatives from these underserved group(s) to understand the barriers they face, and then collaboratively identify service modifications that might improve outcomes. These engagement and co-creation processes should seek to obtain meaningful and actionable data with minimum time and resource requirements.

Our team has conducted a scoping review of rapid qualitative methods that can be used to elicit barriers and potential solutions [23]. Based on this work we have developed a bespoke rapid qualitative elicitation approach: research assistants will perform telephone interviews with non-attenders in each setting and use an a priori deductive framework to code responses. The sample size will be determined by thematic saturation. The long list of barriers and potential solutions derived from these interviews will not necessarily be generalisable to all non-attenders from the same underserved group. To identify the potential solutions that are felt to offer the most value by a statistically representative sample, we will send SMS messages to approximately 400 other non-attenders from the same underserved group, asking them to rank the mooted solutions. The top-ranked interventions will be reviewed by the national leadership team to assess risk, cost, feasibility, and likely impact. Safe and feasible interventions that have a scientifically plausible mechanism of action will be implemented and rigorously evaluated. A detailed protocol for this elicitation process has been published online [24].

#### Testing promising interventions

Once a set of interventions have been derived from engaging with non-attenders, the next step is to implement them and evaluate whether they improve outcomes and reduce sociodemographic gaps. The IM-SEEN model uses a platform randomised controlled trial (RCT) design to assess whether a service modification is causally associated with improvement. This means that the intervention is randomly allocated to individuals or sites. This is only ethical when there is clinical equipoise i.e., it is unclear whether the intervention is better or worse than the status quo. Each intervention will be reviewed by an independent in-country ethics committee.

Allocation, outcome assessment, statistical testing, and reporting should be automated as much as possible to reduce costs to the health programme. Changes within the most underserved groups are the primary outcomes. Mean changes for the entire population is a secondary outcome.

In Botswana’s eye screening programme, we have embedded an automated platform trial that routinely collects and analyses all referral and attendance data. A simple Bayesian algorithm coded in R allocates referred individuals to the intervention or control arm, automatically reviews attendance data, and performs interim statistical testing according to predetermined stopping rules. The algorithm continually adjusts the allocation ratio to favour the best-performing arm(s), minimising the number of people who are assigned to less/ineffective arms. Our trial is not yet complete, but the detailed protocol has been published elsewhere [25].

We are in the process of seeking ethical approval to establish platform RCTs in each country. These use a master protocol that specifies the population (people identified with an eye care need) and primary outcome (attendance), but allow multiple interventions to be tested over time. Every time a new intervention is suggested, ethics committees only have to review the risks of that intervention, having already approved the overall trial architecture. This makes it much more efficient than running serial individual RCTs for each new intervention that is suggested. We are in the process of publishing a detailed protocol for the overall platform trial design.

#### Taking effective service improvements to scale

Once interventions have been rigorously assessed, the final step is to take effective interventions to scale across the entire national programme and then repeat the cycle. We envisage that the process will lead to incremental improvements, with approximately 1–2 cycles per year, depending on local leadership and resourcing.

### Why is this model needed?

#### From data collection to action

Many services now acknowledge and quantify inequalities but do not or cannot translate this intelligence into meaningful action. Where it does happen, the disaggregation of data to assess inequalities and intersectionality [26] often occurs only at the completion of a programme, when there is low potential for the findings to result in change. We feel that there is a need for a practical tool to guide managers through the process of systematically analysing routinely collected sociodemographic data in real-time, and then turning that insight into robust action to improve outcomes for all service beneficiaries, with the greatest effort focused on those with the greatest need.

#### Engaging and co-creating

Whilst people affected by a given problem tend to have sensible ideas about how to fix it, initiatives to target underserved groups (e.g. those living in remote areas) are rarely developed with meaningful input from service users themselves [27, 28]. Instead, managers sit down to discuss potential issues and solutions on behalf of the underserved groups, and then implement service modifications without further consultation. This is partly because it can be time-consuming and expensive to seek non-tokenistic input from others – especially from those at the margins of society [27]. However, this needs to change. Community engagement and empowerment is one of the core tenets of Primary Health Care [29] and all governments have committed to deliver health systems that place greater decision-making power in the hands of the people [9, 29].

A model for continuous equity-driven service improvement should meaningfully engage with representatives of the groups found to be facing the highest barriers. Ultimately it is these service users who have the best understanding of why they cannot access care or achieve good outcomes, and they are likely to have practical ideas for how the service could be modified to better serve their population.

We note that service leaders need scientifically robust yet rapid and affordable methods for eliciting barriers and co-designing solutions, however current engagement exercises tend to cluster between two opposing poles: expensive, bespoke, in-depth qualitative research that takes many months to plan and execute on one hand, and zero/tokenistic engagement on the other. The first approach provides robust findings at a very high cost for service providers, the second is affordable but does not produce usable intelligence. Somewhere between the two lies a minimum viable product; the cheapest and fastest possible approach that delivers meaningful data based on genuine engagement.

Industry tends to use focus groups and telephone surveys for rapid market research, but we are not aware of any rapid pragmatic research methods being routinely used in health service improvement; for instance, the recent King’s Fund workshop on ‘improving services by listening to patient voices’ did not showcase any qualitative methods that could be conducted in fewer than six months [30]. This is a strategic barrier to co-production [31]. Our work to develop rapid yet robust methods represents a step forward, but our approach is still in the process of being tested. The IM-SEEN model stipulates that ideas for service improvements should come from engagement with affected communities, but does not dictate the exact methods as different contexts require different approaches.

#### Checking whether ‘service improvements’ actually improve services

Once potential solutions have been identified it is vital that they are rigorously evaluated. This should entail checking whether any changes made to the service lead to changes in outcomes – positive or negative – as well as understanding the effect size and distribution among different groups. Specifically, it is important to check that access and outcomes improve for all groups, ideally with the greatest gains observed among groups with the greatest need.

Despite widespread lip service to ‘continuous improvement’, in our experience, service modifications designed to boost equity are often conducted as one-off initiatives. Furthermore, efforts to reduce inequalities tend to be poorly evaluated [10]. This is surprising given the rise and rise of *Plan Do Study Act* cycles [32–34]. Whilst the core ‘PDSA’ model is based on the scientific approach of formulating a hypothesis, collecting data to test the hypothesis, analysing and interpreting results, and making inferences to iterate the hypothesis, [35] most quality improvement initiatives fail to quantify change appropriately and it is rare to find truly iterative examples where services have progressed through more than one or two revolutions of the cycle [36, 37].

Even when a service *does* routinely gather high quality data and test hypothesis-driven innovations, the process tends to be limited by an overdependence on crude before-after testing or interviews with a handful of service users (which can offer valuable information about how/why and intervention works but tells us nothing about the mean effect size). We need to be sure that any observed changes in outcomes are driven by service modifications. More than that, we need to ask if it is ethical to modify services without recourse to robust means of evaluating impact – especially where unintended consequences could lead to harm or a deterioration in service quality or equity.

The most robust means of evaluating whether service innovations, reconfigurations, amendments, adaptations, and other ‘improvements’ actually confer benefit is by conducting randomised controlled trials [38]. However, RCTs are generally expensive, require specialist statistical support, and can take years to run, rendering them unfeasible for most settings [39]. When resources are available, the expensive price tag exerts a strong pressure to reserve this tool for service amendments that have a high ‘pre-test’ probability of success. This means that the least robust service modifications are systematically subjected to the weakest levels of methodological scrutiny, potentially squandering resources, incurring opportunity costs, and even exposing users to harm.

The rising use of RCTs in industry – often referred to as ‘A/B testing’—has spawned a wave of low-cost, real-time, automated approaches to running real-time pragmatic trials in order to optimise services with high-quality empirical data. The ‘test everything with controlled experiments’ approach was born of the observation that tiny service changes sometimes had large impacts on important outcomes, and that most large, expensive reforms based on promising ideas fail to deliver the intended change [40]. Allied work from non-health areas of continuous improvement has demonstrated that multiple small improvements can lead to large overall gains – strengthening the case for multiple rapid tests of multiple service modifications [41, 42]. This mature and powerful ‘test everything’ approach is being used to optimise search engines, improve web page click-throughs, and drive profit margins [43–45] but has not yet made the transition to health service improvement.

As health programmes increasingly digitise patient flow, opportunities are emerging to embed prospective randomisation and statistical testing into administrative software [46]. The adoption of ‘built-in’ testing would reduce the barriers for routine RCT testing. By making it easier to perform RCTs to test service modifications, we would vastly improve safety by helping managers to reliably differentiate between effective and ineffective amendments. The automation of randomization, allocation, and statistical analysis works best when algorithms can be directly embedded into clinical software, as this eliminates the delays associated with human factors.

Even automated RCTs still take time and specialist expertise to set up, and these costs mean that programmes will have fewer resources to deploy for service delivery. The time taken to design the trial and obtain ethical approval can also delay the implementation of potential service improvements. These ethical issues must be weighed against the fact that introducing interventions without robust evaluation can lead to the unknowing delivery of ineffective or harmful interventions. Nevertheless, given the work, time and costs involved in setting up a platform trial, this approach will deliver the greatest cost-benefits if used to continually assess a large number of interventions over a long period of time.

Changes and interventions that are found to be effective at improving outcomes and reducing the inequalities should be taken to scale across entire services. In summary, there is a need to develop embedded RCT testing code that can run resource-light trials in order to provide robust evidence on whether well-intentioned service modifications are helping or harming.

## Discussion

In this paper we have presented an overview of the IM-SEEN model and a description of how we are applying it in the field of eye health in four different country programmes. A key strength and limitation of the model is that is describes essential elements but does not prescribe the exact methods. Whilst we are using a specific set of sociodemographic indicators and multivariable logistic regression to identify groups with the lowest attendance rates in Botswana, Kenya, India and Nepal, this specific approach will not be appropriate for all scenarios. To take a hypothetical example, a regional cervical screening service associated with urban/rural disparities may want to use chi-square testing, followed by Rapid Anthropological Assessment [47] as these specific methods are best suited to the programme’s needs. Similarly, our model is based on the use of automated adaptive RCTs as these minimise the number of people exposed to ineffective or harmful interventions and should facilitate rigorous and efficient continuous identification of service modifications that improve equitable outcomes. However, there are virtually infinite potential configurations for these RCTs and it would not be appropriate for our team to mandate one specific approach.

Whilst the model is been designed for use in any field, its initial deployment and empirical testing is underway in community-based eye health services. Our model directly supports the recommendations of the 2019 World Report on Vision through promoting high quality implementation and health systems research, empowering people and communities, and creating an enabling environment to implement integrated people centred eye care [48]. These themes resonate with the core pillars of the Astana Declaration on Primary Health Care: empowering people and communities, and advancing equitable care that is responsive to local needs [29].

One major advantage of testing the model in smartphone-based eye screening programmes is that exposure and outcome data are routinely digitally collected and stored in a unified database where an automated testing system can operate with minimal need for human intervention. We are keen to apply the model to address other areas such as the inequitable uptake of cancer screening, inequitable diagnosis and provision of treatment for diabetes and hypertension, and the distribution of vaccines. The model demands that sociodemographic data are obtained from intended service beneficiaries and that the primary outcome is recorded – be that attendance, treatment, cure, or anything else. Ideally, the primary outcome will be recorded routinely and digitally for every patient. Where this is not the case, additional costs will be incurred. Taking eye care as an example, the ultimate outcome is corrected vision but service attendance is often used as a proxy.

There has been a proliferation of theoretical models of proportionate universalism and pro-equity service delivery, but as Francis-Oliviero and colleagues note in their review of the field, interventions and real-world examples are rare [10]. As far as we are aware, the IM-SEEN model is the first operational model that has been developed to drive continuous evidence-based and equitable improvement in real-world programmes. As results from the model’s application in the field of eye care services emerge, we will continue to refine the approach and apply it to other areas. We encourage other researchers, programme managers and policymakers to adopt the principles – if not the model itself in future work to extend health service coverage to all groups, with a focus on those with the greatest need.

## Data Availability

No data are available.
